# Liking the Look, but Looking Less? Relative Evaluative Compatibility and Early Orienting to Opposite-Gender Beautified Faces

**DOI:** 10.3390/bs16081421

**Published:** 2026-08-18

**Authors:** Linzhe Cheng, Xiangfei Liu, Yawen Zhao, Xia Wu

**Affiliations:** 1 Faculty of Psychology, Tianjin Normal University, Tianjin 300387, China; 13333442537@163.com (L.C.); 15027477384@163.com (X.L.); 19835102399@163.com (Y.Z.); 2 Key Research Base of Humanities and Social Sciences of the Ministry of Education, Academy of Psychology and Behavior, Tianjin 300387, China; 3 Tianjin Social Science Laboratory of Students’ Mental Development and Learning, Tianjin 300387, China

**Keywords:** beautified faces, attentional bias, evaluative compatibility, social media

## Abstract

The widespread use of beauty-enhancement technology has raised questions about how beautified faces are evaluated and prioritized during attentional processing. This study combined an Implicit Association Test (IAT) and a dot-probe task to examine relative evaluative compatibility and attentional responses to beautified and non-beautified (original) synthetic same-gender (female) and opposite-gender (male) target faces among 45 heterosexual female university students. The results showed greater relative compatibility for the beautified-positive/original-negative mapping than for the reversed mapping, while evidence that this compatibility effect varied by target face gender remained inconclusive. Furthermore, participants showed early attentional orienting toward opposite-gender beautified faces at 100 ms, whereas the reversed validity pattern at 500 ms was not statistically significant. Additionally, the opposite-gender D-score was negatively associated with the opposite-gender 100 ms attention-bias index. Given the well-documented psychometric constraints of reaction-time difference scores in dot-probe tasks, this individual-difference association should be treated as preliminary and requires further replication. In the present sample, the direction of this association was compatible with a habituation-based account, although exposure history and habituation were not directly assessed. These findings provide evidence concerning relative evaluative compatibility and early attentional responses to beautified versus non-beautified synthetic faces in heterosexual female university students.

## 1. Introduction

The pursuit of beauty is a widespread human tendency. In contemporary digital media environments, however, this pursuit has increasingly shifted toward “beauty enhancement technology”—sophisticated software that algorithmically reconstructs portraits to generate idealized, high-attractiveness faces (Rathgeb et al., 2019). For many users, particularly younger users, digital retouching has evolved into a strategic form of “photo investment” used to gain social recognition and shape self-presentation (Fu & Luo, 2023). Yet, this technological intervention has raised concerns about the tension between aesthetic standardization and authentic identity. Critics argue that the algorithmic homogenization of beauty promotes a mechanized look that systematically obscures the “truth” of individual features (Hussain et al., 2025; Lee & Lee, 2021; Miller, 2026). This conflict has already manifested in significant societal repercussions; for instance, the recent decision by postgraduate entrance exam systems to ban “Haima-style” (highly retouched) photographs underscores a growing institutional concern that excessive beautification “veils reality” and leads to identification failures (IT Times, 2024). Such phenomena suggest a deeper cognitive shift: as aesthetics are reshaped by algorithmic logic, individuals may be paying less attention to unaltered facial features (Rankin et al., 2009). Despite the intensity of these public debates, empirical evidence remains scarce regarding whether prolonged exposure to such idealized digital imagery has fundamentally altered the underlying implicit evaluations and attentional priorities that govern human social perception.

This potential cognitive shift may be reflected in the relationship between implicit evaluations and attentional allocation (Posner & Petersen, 1990; Cisler & Koster, 2010). Theoretically, an individual’s evaluative tendency toward a stimulus—their implicit attitude—may function as an implicit value signal that influences processing priority (Anderson, 2021). When encountering beautified faces, these automatically activated associations are thought to govern bottom-up attentional selection, influencing how rapidly a face attracts initial attention and how long it sustains attentional engagement (Posner & Petersen, 1990). To accurately capture these dynamics, it is essential to move beyond self-report measures, which are often confounded by impression management or limited by a lack of introspective access (Nosek et al., 2011). Implicit measures can instead assess evaluative tendencies that operate with minimal intentional control or conscious awareness (Greenwald & Lai, 2020; Forscher et al., 2019). This approach is particularly pertinent in the context of social media’s algorithmic feeds, where the rapid, low-effort browsing of imagery means that social-cognitive responses may be strongly influenced by these spontaneous appraisals rather than deliberate reflection (Payne et al., 2017).

Within this theoretical framework, two competing mechanisms offer divergent predictions regarding how implicit evaluations govern attentional selection. On the one hand, the incentive-salience perspective posits that positive implicit attitudes should trigger automatic attentional capture, as beautified faces serve as personally salient, reward-linked cues (Posner & Petersen, 1990; Cisler & Koster, 2010). This view aligns with accounts of selection history and value-based priority, suggesting that exposure to reward-linked stimuli biases initial orienting (Anderson, 2021; Zhao et al., 2022; Bai et al., 2023). On the other hand, a habituation account predicts that repeated exposure can reduce the novelty and attentional priority of a stimulus (Rankin et al., 2009). In social-media environments, repeated encounters with idealized imagery may be amplified by users’ content choices and recommendation systems, potentially weakening early orienting toward beautified faces. Under this account, a more positive evaluative tendency could coexist with reduced attentional capture because repeated exposure may gradually diminish the novelty of the stimulus. Resolving whether positivity enhances or inhibits attentional capture is crucial for delineating the long-term cognitive consequences of beauty-enhancement technologies for visual attention.

The proposed relationship between implicit evaluation and attentional selection may also vary according to target face gender, as different faces activate distinct motivational systems that assign varying degrees of cognitive priority. For heterosexual women, opposite-gender (male) faces primarily engage mating-related motives, where facial attractiveness serves as a high-priority signal of “good genes” and reproductive viability (Maner et al., 2003; Shin et al., 2018; Pátková et al., 2025). Within this evolutionary framework, beautified male faces act as potent, reward-linked cues that are expected to command immediate, bottom-up attentional capture. Conversely, same-gender (female) faces may be more likely to elicit social-comparison or identity-related processes, as women may evaluate these faces against internalized aesthetic standards or view them as desired outcomes of appearance-related self-transformation (Mafra et al., 2020; Hussain et al., 2025). These differing motivational drivers—mating attraction versus social appraisal—determine the functional value weight assigned to beautified features, thus dictating whether greater relative evaluative compatibility translates into enhanced attentional orienting or, as the habituation hypothesis suggests, a possible reduction in attentional capture following repeated exposure. Consequently, target face gender may shape how beautified facial features are evaluated and prioritized during attentional selection in heterosexual women.

To investigate these social-cognitive processes in a population for whom beauty-enhancement technologies are particularly relevant, the present study focused on heterosexual female university students. Beauty-enhancement software and appearance-focused social media activities are particularly common among young women (iDigital, 2024; Fu & Luo, 2023). Recent experimental work with women aged 18–30 has also shown that using slimming beauty filters can affect body-image evaluations and self-objectification (Schroeder & Behm-Morawitz, 2025). Restricting the sample to heterosexual women also allowed male target faces to be defined consistently as opposite-gender, potentially mate-relevant stimuli, while female target faces served as same-gender stimuli. This sampling strategy was intended to provide a theoretically coherent test within a specific population rather than to represent women or social-media users more broadly. By integrating the Implicit Association Test (IAT) and the dot-probe task, the present study examined whether relative evaluative compatibility toward digitally beautified faces was associated with attentional selection across early and later processing stages (Anderson, 2021; Greenwald & Lai, 2020). The principal contribution of this study lies in linking these evaluative and attentional responses while separately examining same-gender (female) and opposite-gender (male) target faces. The study further evaluated whether the association between evaluative compatibility and early attentional orienting was more consistent with incentive-salience or habituation-based predictions. It was therefore designed to characterize evaluative and attentional responses within this specific sample and experimental context.

Based on the theoretical framework outlined above, we propose the following hypotheses:

**H1.**  *Consistent with the “what is beautiful is good” stereotype, participants will show greater relative compatibility for the beautified-positive/original-negative mapping than for the reversed mapping.*

**H2.**  *We hypothesize that target face gender will moderate the effect of beautification on attentional allocation. Specifically, driven by mating-related motives and the signaling of “good genes”, opposite-gender (male) beautified faces are expected to elicit stronger early attentional orienting than same-gender beautified faces, which are expected to primarily elicit social-comparison processes.*

**H3.**  *We hypothesize that gender-specific evaluative compatibility will be associated with early attentional bias. A positive association would be consistent with the incentive-salience perspective, whereas a negative association would be consistent with a habituation-based account.*

## 2. Materials and Methods

### 2.1. Participants

The sample size for the 2 × 2 within-subjects design was calculated using MorePower 6.0 (Campbell & Thompson, 2012), with α = 0.05, an effect size of η^2^ = 0.30, and statistical power of 0.90, resulting in a required sample size of 28. To allow for potential participant attrition, invalid data, or equipment malfunction, 45 heterosexual female university students aged 18–24 years (M = 19.89 years, SD = 1.22) were recruited from Tianjin Normal University in northern China. All participants were Han Chinese university students. No further information regarding ethnic subgroups or regional cultural backgrounds was collected. The recruitment notice specified heterosexual female university students as the eligible population, and eligibility regarding sexual orientation was based on participants’ self-report during recruitment rather than on a standardized measure. All participants reported being in good physical health and having normal or corrected-to-normal vision. They provided informed consent before the experiment and received compensation upon completion. The study procedures and protocols were approved by the Ethics Committee of Tianjin Normal University (protocol code 2025030311; date of approval: 3 March 2025). All 45 participants completed both tasks and were included in the analysis.

### 2.2. Procedure

#### 2.2.1. Experimental Materials

Implicit Association Test (IAT) Image Materials: The baseline stimuli were synthetic face images obtained from an AI-generated-face website (https://www.thispersondoesnotexist.com/, accessed on 29 April 2024), including five Asian male and five Asian female faces. These non-beautified images are referred to as “original faces” throughout the manuscript to distinguish them from their subsequently beautified versions; they were not photographs of real individuals. All facial images used in the manuscript were based on fully AI-generated synthetic faces rather than photographs of real individuals; therefore, no copyright permission was required for the depicted faces. Beautified versions of these images were generated by the research team before the experiment using the default built-in “one-click beautification” function in Meitu XiuXiu (version 10.20.5). The same preset was applied uniformly to all facial images, with no manual adjustment of sliders, local retouching, or customized beautification intensity. The proprietary one-click function does not provide end users with access to specific computational procedures, feature-weighting parameters, or facial-transformation settings underlying the preset; therefore, the magnitude of changes to individual facial features could not be separately quantified. Visual inspection indicated that non-facial elements, including hairstyle, clothing, and background, remained unchanged between the original and beautified versions.

**Figure 1:**
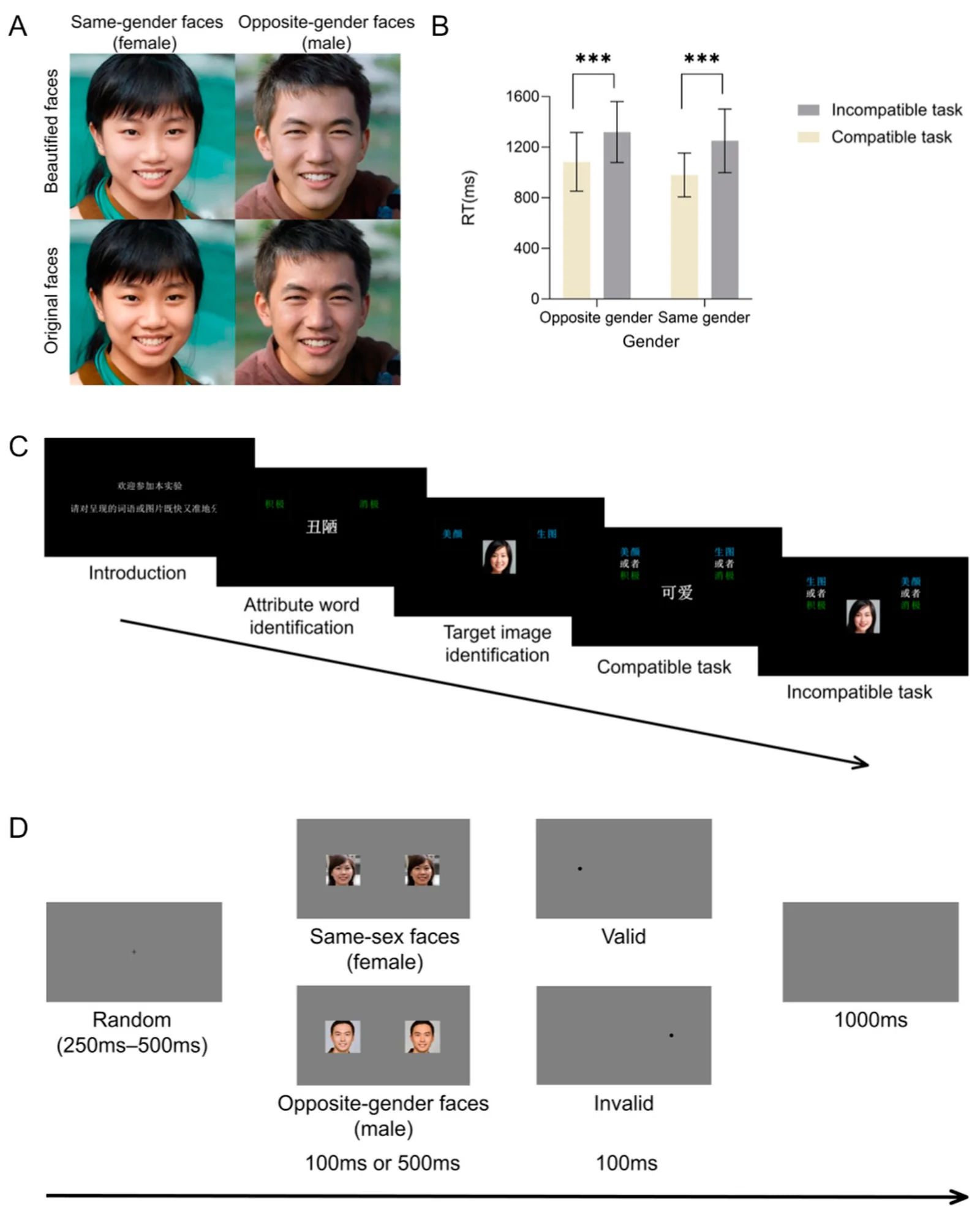
(**A**) Examples of beautified and original (non-beautified) synthetic face stimuli in the same-gender (female) and opposite-gender (male) conditions. (**B**) Mean response times (M ± SD) across task types and target face genders in the IAT. *** *p* < 0.001. Error bars represent standard deviations. (**C**) IAT procedure. The arrow indicates the sequence of the task. The Chinese instruction means “Please classify the presented words or images as quickly and accurately as possible”, while the remaining Chinese terms correspond to the positive and negative attribute words listed in Section 2.2.1. (**D**) Dot-probe task procedure.

Dot-Probe Task Image Materials: The baseline stimuli consisted of 56 Asian male and 39 Asian female synthetic face images obtained from the same website. Each image was processed using the same default built-in “one-click beautification” function in Meitu XiuXiu (version 10.20.5), following the same standardized procedure described above, with no manual adjustment of sliders, local retouching, or customized beautification intensity. This procedure produced a beautified version paired with its corresponding original version (examples are shown in Figure 1A). Additionally, 163 female university students, who were not part of the main experiment, rated the attractiveness of the faces on a 9-point scale. Ultimately, 25 male and 25 female face pairs were selected. Paired-sample *t*-tests confirmed that, within each selected pair, the beautified face was rated as more attractive than the corresponding original face (see Supplementary Materials).

IAT Words: In total, 31 female university students (M = 20.06 years, SD = 1.22), who were not part of the main experiment, freely described “beautified” and “original” faces, yielding 18 positive and 18 negative words. Additionally, 30 other female university students (M = 21.23 years, SD = 4.81), who were not part of the main experiment, selected the 10 positive and 10 negative words judged to be most relevant to the “original” and “beautified” face categories. Finally, the words were ranked by selection frequency, resulting in the following:

Positive Words: Exquisite, Beautiful, Delicate, Vibrant, Natural, Attractive, Pure, Fresh, Cute, Lively.

Negative Words: Artificial, Terrible, Stiff, Exaggerated, Grotesque, Decadent, Pretentious, Ugly, False, Sloppy.

#### 2.2.2. Experimental Procedure

All stimuli were presented on a Lenovo 14-inch monitor (60 Hz; resolution 2880 × 1800), with participants seated at a viewing distance of approximately 62 cm. Presentation and data acquisition were controlled with E-Prime 2.0.10. Participants completed both the Implicit Association Test (IAT) and the dot-probe task, with task order counterbalanced across participants.

Implicit Association Test (IAT):

The IAT task used a 2 (task type: compatible and incompatible) × 2 (target face gender: same gender and opposite gender) within-subjects design. In the compatible task, the response keys were consistent with pairing beautified faces with positive words and original faces with negative words; in the incompatible task, the response keys were reversed, pairing beautified faces with negative words and original faces with positive words. The target face gender categories were same-gender (female) and opposite-gender (male). Specifically, same-gender and opposite-gender faces were presented in a pseudo-random order within the experimental blocks, and participants were required to categorize them based solely on the “beautified” versus “original” dimension, regardless of face gender.

Before beginning the task, participants familiarized themselves with the target face categories (beautified and original) and the attribute categories (positive and negative).

The IAT used a seven-block procedure adapted from Greenwald et al. (2003), as summarized in Table 1. The stimuli were presented at the center of the screen against a black background. Face stimuli subtended approximately 3° × 3°, and word stimuli subtended approximately 1.97° × 1.17°.

Block 1 consisted of 20 attribute-discrimination trials in which participants categorized positive and negative words. Block 2 consisted of 20 target-discrimination trials in which participants categorized beautified and original faces. Blocks 3 and 4 comprised the compatible combined task. During the practice block (Block 3, 20 trials) and the critical test block (Block 4, 60 trials), beautified faces and positive words shared one response key, whereas original faces and negative words shared the other response key.

Block 5 consisted of 20 reversed target-discrimination trials, in which the response-key assignments for beautified and original faces were reversed. Blocks 6 and 7 comprised the incompatible combined task. During the practice block (Block 6, 20 trials) and the critical test block (Block 7, 60 trials), beautified faces and negative words shared one response key, whereas original faces and positive words shared the other response key.

The order of the compatible and incompatible combined-task sequences was counterbalanced across participants. Half of the participants completed the compatible sequence before the incompatible sequence, whereas the remaining participants completed the two sequences in the reverse order. The attribute-discrimination block was completed first by all participants. Feedback was provided during the practice blocks, and each trial continued until a response was registered. The IAT comprised 220 trials and lasted approximately 8 min. The experimental procedure is illustrated in Figure 1C.

Dot-Probe Task:

To investigate the time course of attentional bias toward beautified faces, we adopted the standard dot-probe paradigm (MacLeod et al., 1986). The dot-probe task used a 2 (target face gender: same gender and opposite gender) × 2 (validity: valid and invalid) × 2 (SOA: 100 ms, 500 ms) within-subjects experimental design. The 100 and 500 ms SOAs were selected following established facial dot-probe research examining attentional allocation at brief and longer stimulus durations (Cooper & Langton, 2006). The procedure is shown in Figure 1D. The task background was gray (RGB: [127, 127, 127]). At the beginning of each trial, a black “+” fixation cross was presented at the center of the screen for a randomly selected duration between 250 and 500 ms. After the fixation cross disappeared, a pair of 3° × 3° face images, consisting of one beautified face and its corresponding original version, was presented simultaneously to the left and right of fixation for either 100 or 500 ms. Thus, the face-presentation duration was identical to the stimulus onset asynchrony (SOA) between face onset and probe onset. Immediately after the face pair disappeared, with no intervening blank interval, a black dot probe (0.5° × 0.5°) appeared for 100 ms at one of the two locations previously occupied by the faces. Participants were required to indicate the probe location as quickly and accurately as possible by pressing the F key for a left-side probe and the J key for a right-side probe. Responses were accepted for up to 1000 ms from probe onset. The trial was considered valid if the probe appeared in the same location as the beautified face, and invalid if it appeared in the location of the original face. The experimental trials were fully randomized for each participant. Target face gender, SOA, validity, and the left–right position of the beautified face were balanced across trials. The experiment included 32 practice trials using a separate set of stimuli that was not used in the main experiment and 400 experimental trials (with a break after every 134 trials), lasting approximately 15 min.

### 2.3. Data Analysis

Data processing and frequentist statistical analyses were conducted using SPSS (Version 23.0.0.0), and Bayesian analyses were conducted using JASP (Version 0.98.21.0). The Bayes factor (BF_10_) quantified the relative evidence for the alternative hypothesis over the null hypothesis. Values greater than 1 favored the alternative hypothesis, whereas values below 1 favored the null hypothesis, with the magnitude of the Bayes factor indicating the strength of evidence (Wagenmakers et al., 2018). For the Bayesian ANOVAs, inclusion Bayes factors (BF_incl_) were reported to compare the evidence for models including a particular effect with evidence for models excluding that effect. Values above 1 favored inclusion of the effect, whereas values below 1 favored its exclusion; values close to 1 were interpreted as providing only limited evidence in either direction (Van den Bergh et al., 2020). For the IAT, trials with response times shorter than 300 ms or longer than 10,000 ms were excluded, and participants with an overall error rate exceeding 20% were excluded. Incorrect trials that met the response-time criteria were assigned the mean correct response time of the corresponding block plus a 600 ms penalty (Greenwald et al., 2003; Hendriks et al., 2022; Zhi et al., 2024).

Gender-specific D-scores were calculated using the two critical combined blocks (Blocks 4 and 7). Each block contained 20 male-face trials, 20 female-face trials, and 20 attribute-word trials. For the gender-specific analyses, the attribute-word trials were duplicated during data restructuring and included once in both the male-face and female-face subsets. Thus, each gender-specific subset contained 20 gender-specific face trials and 20 attribute-word trials per block. For each subset, the mean response time in the compatible condition was subtracted from that in the incompatible condition, and the resulting difference was divided by the standard deviation of all corrected response times pooled across the two blocks. Larger positive D-scores indicated greater relative compatibility of the beautified-positive/original-negative mapping than of the reversed mapping (Greenwald et al., 2003; Karpinski & Steinman, 2006). Because the same attribute-word trials contributed to both subsets, the same-gender and opposite-gender D-scores were treated as condition-specific decompositions of the overall IAT effect rather than as fully independent measures.

Processed response times were analyzed using a 2 (task type: compatible and incompatible) × 2 (target face gender: same gender and opposite gender) repeated-measures ANOVA. The internal consistency of each gender-specific D-score was evaluated using 10 balanced trial parcels. To assess the dependence of the estimates on a particular allocation of trials, the balanced parceling procedure was repeated 5000 times.

For the dot-probe task, incorrect and nonresponse trials were excluded from the response-time analysis, as were correct trials with response times more than three standard deviations above the participant-specific mean. Given well-documented challenges in estimating reliable difference-score indices (e.g., Schmukle, 2005), group-level validity effects were emphasized, whereas analyses treating the attention-bias index as an individual-difference measure were considered preliminary. Accuracy and response times were analyzed separately using 2 (target face gender: same gender and opposite gender) × 2 (validity: valid and invalid) × 2 (SOA: 100 ms, 500 ms) repeated-measures ANOVAs. Attention-bias indices were calculated by subtracting mean response times on valid trials from those on invalid trials. Pearson correlations were conducted between the two gender-specific D-scores and the four attention-bias indices. The resulting eight D–BI correlations were adjusted using the Benjamini–Hochberg false discovery rate procedure, and the focal significant association was further examined using simple linear regression. The significance level was set at *p* < 0.05.

## 3. Results

### 3.1. Implicit Association Test

No participant exceeded the 20% overall IAT error-rate criterion. The overall error rate was 4.28%. Two incorrect trials also met the response-time exclusion criteria and were therefore excluded, whereas the remaining incorrect trials were retained after application of the 600 ms penalty described in Section 2.3.

The repeated-measures ANOVA on processed response times (Figure 1B) revealed a significant main effect of task type: F(1, 44) = 86.502, *p* < 0.001, ηp^2^ = 0.663. Responses were faster in the compatible task (1032.67 ± 27.51 ms) than in the incompatible task (1285.19 ± 35.29 ms). The main effect of target face gender was also significant, F(1, 44) = 17.412, *p* < 0.001, ηp^2^ = 0.284, with faster responses to same-gender faces (1115.83 ± 29.31 ms) than to opposite-gender faces (1202.01 ± 31.44 ms). However, the task type × target face gender interaction did not reach statistical significance: F(1, 44) = 2.535, *p* = 0.119. The corresponding inclusion of the Bayes factor, BF_incl_ = 2.508, provided limited support for models, including the interaction. Taken together, the frequentist and Bayesian results did not permit a firm conclusion regarding whether the compatibility effect varied by target face gender.

The opposite-gender D-score had a Cronbach’s alpha of 0.714, whereas the same-gender D-score had an alpha of 0.586. Across 5000 repetitions of the balanced parceling procedure, the mean alpha coefficients were 0.702 and 0.558, respectively. Thus, the opposite-gender D-score showed acceptable internal consistency, whereas the same-gender D-score showed more modest internal consistency and was interpreted cautiously.

### 3.2. Dot-Probe Task

**Figure 2:**
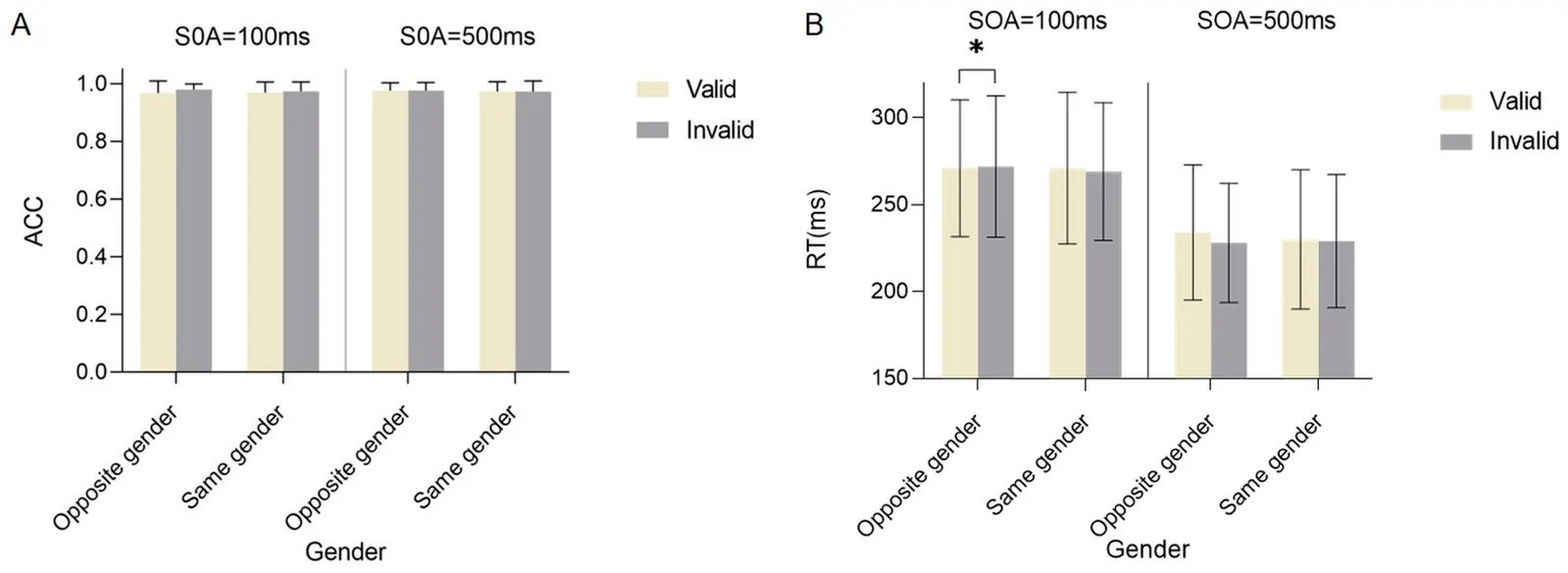
(**A**) Accuracy rates (M ± SD) across conditions in the dot-probe task. (**B**) Response times (M ± SD) under different conditions in the attentional bias task. *: *p* < 0.05. Error bars represent standard deviations.

A 2 (target face gender: same gender and opposite gender) × 2 (validity: valid and invalid) × 2 (SOA: 100 ms, 500 ms) repeated-measures ANOVA was conducted on accuracy rates. The results (Figure 2A) showed that the main effect of target face gender was not significant: F(1, 44) = 2.208, *p* = 0.144, BF_incl_ = 0.122. The main effect of validity was also not significant: F(1, 44) = 1.733, *p* = 0.195, BF_incl_ = 0.183. The main effect of SOA was not significant: F(1, 44) = 0.570, *p* = 0.454, BF_incl_ = 0.107.

The interaction between target face gender and validity was not significant: F(1, 44) = 0.523, *p* = 0.474, BF_incl_ = 0.049. The interaction between target face gender and SOA was not significant: F(1, 44) = 0.089, *p* = 0.767, BF_incl_ = 0.027. The interaction between validity and SOA was not significant: F(1, 44) = 2.507, *p* = 0.120, BF_incl_ = 0.107. The three-way interaction between target face gender, validity, and SOA was not significant: F(1, 44) = 0.564, *p* = 0.457, BF_incl_ = 0.003.

In the dot-probe task, 2.65% of trials were incorrect or contained no response and were excluded from the reaction-time analysis. Among the correct trials, a further 1.45% were excluded because their response times exceeded the participant-specific mean by more than three standard deviations. The reaction-time analysis (Figure 2B) showed that the main effect of target face gender was not significant: F(1, 44) = 0.002, *p* = 0.963, BF_incl_ = 0.070. The main effect of validity was also not significant: F(1, 44) = 0.399, *p* = 0.531, BF_incl_ = 0.132. The main effect of SOA was significant: F(1, 44) = 328.708, *p* < 0.001, ηp^2^ = 0.882. Response times in the 500 ms SOA condition (236.58 ± 5.56 ms) were significantly faster than those in the 100 ms SOA condition (278.05 ± 5.98 ms).

The three-way interaction among target face gender, validity, and SOA was significant: F(1, 44) = 5.372, *p* = 0.025, ηp^2^ = 0.109. Follow-up analyses were conducted to clarify this interaction. At 100 ms, the validity effect was significant for opposite-gender faces, F(1, 44) = 5.807, *p* = 0.020, ηp^2^ = 0.117, with faster responses on valid trials than on invalid trials (276.13 ± 5.86 ms vs. 280.38 ± 6.07 ms). No significant validity effect was observed for same-gender faces: F(1, 44) = 0.878, *p* = 0.354, BF_10_ = 0.244. A direct comparison of the attention-bias indices showed that the validity effect was significantly larger for opposite-gender than for same-gender faces: t(44) = 2.33, *p* = 0.024, d_v_ = 0.35. At 500 ms, responses on valid trials were numerically slower than those on invalid trials for opposite-gender faces (238.02 ± 5.80 ms vs. 234.64 ± 5.12 ms), but this contrast did not reach statistical significance: F(1, 44) = 3.726, *p* = 0.060, ηp^2^ = 0.078. The validity effects for opposite-gender and same-gender faces did not differ significantly at 500 ms: t(44) = −0.90, *p* = 0.373, d_v_ = −0.13. The reversed validity pattern observed for opposite-gender faces at 500 ms may be compatible with inhibition of return, but this interpretation should be treated cautiously because the relevant contrast was not statistically significant.

### 3.3. Relationship Between Evaluative Compatibility and Attentional Bias

For ease of interpretation, larger positive D-scores indicated greater relative compatibility for the beautified-positive/original-negative mapping than the reversed mapping, whereas positive BI values indicated faster responses when the probe replaced the beautified face (Greenwald et al., 2003; Karpinski & Steinman, 2006; Tran et al., 2013). The two gender-matched correlations at 100 ms—between the same-gender D-score and the same-gender BI, and between the opposite-gender D-score and the opposite-gender BI—served as the primary hypothesis-guided tests derived from H3. The remaining six D–BI correlations were examined exploratorily.

**Figure 3:**
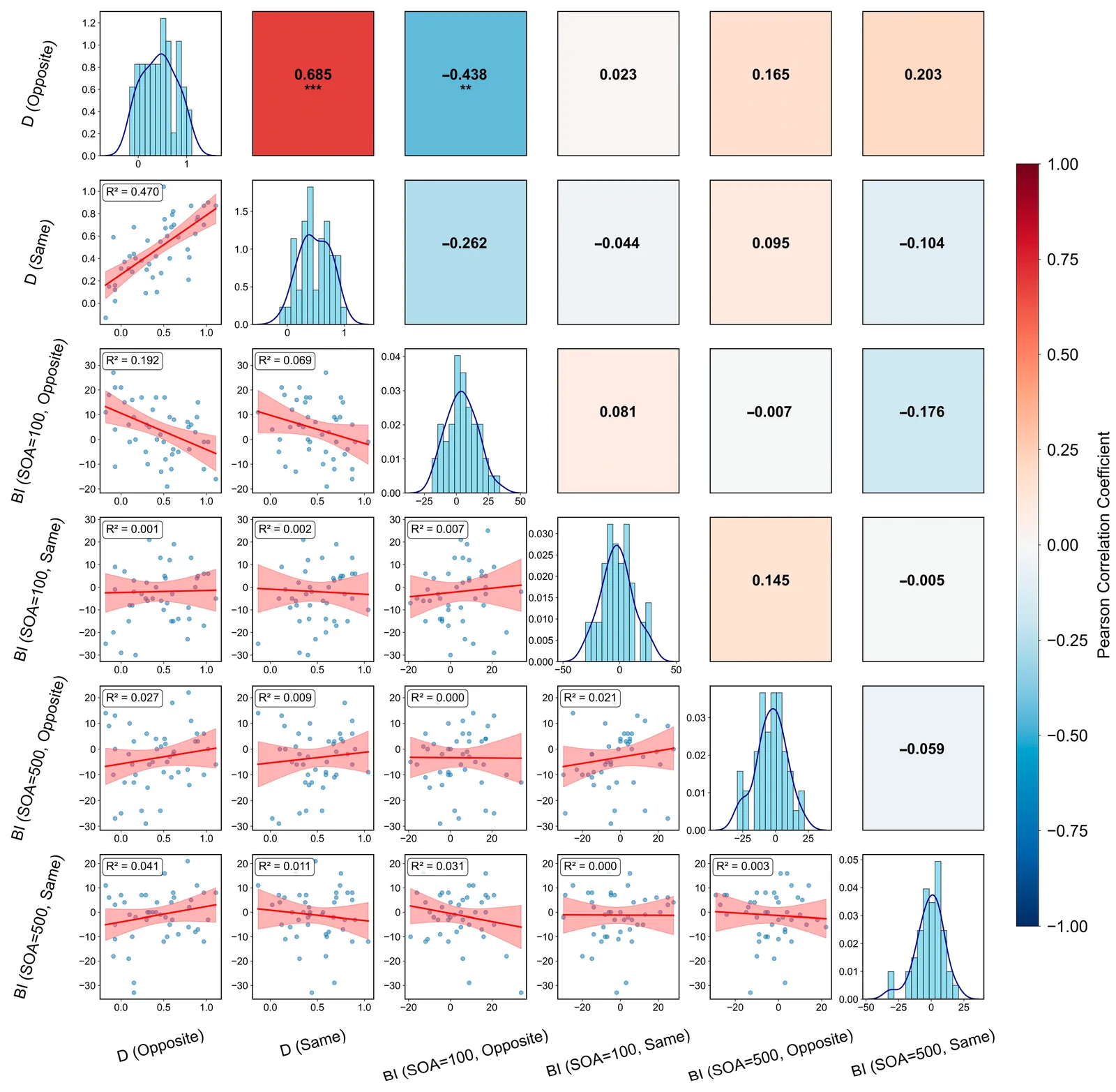
Pearson correlation matrix for the two D-scores and four attention-bias indices. In the upper triangle, cell colors indicate the direction and magnitude of Pearson correlations (red = positive; blue = negative). In the lower triangle, blue circles represent individual participants, and red lines represent fitted linear regression lines. The blue curves along the diagonal show the distributions of each variable. Asterisks indicate unadjusted two-tailed *p*-values (** *p* < 0.01, *** *p* < 0.001).

Among the eight D–BI associations, the opposite-gender D-score was negatively correlated with the opposite-gender BI in the 100 ms condition: r = −0.44, *p* = 0.003, FDR-adjusted *p* = 0.021. None of the remaining D–BI correlations reached statistical significance after correction (all FDR-adjusted *p*s > 0.05). Figure 3 presents the full correlation matrix for descriptive completeness; correlations between the two D-scores and among the four BI scores were not treated as additional hypothesis tests.

**Figure 4:**
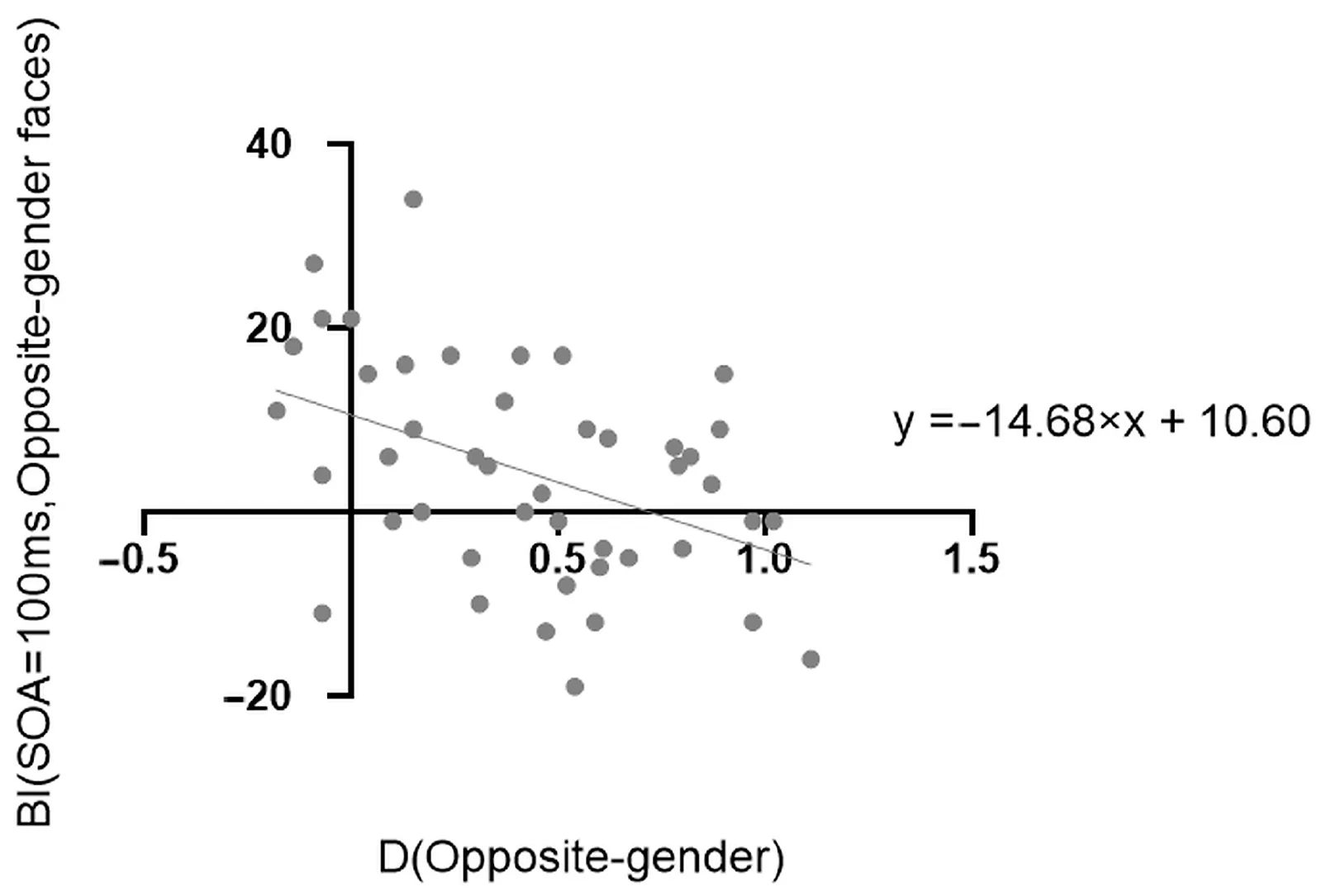
Association between the opposite-gender D-score and the opposite-gender 100 ms attention-bias index. Grey dots represent individual participants, and the grey line represents the fitted linear regression line.

The corresponding simple linear regression model was significant: F(1, 43) = 10.25, *p* = 0.003, R^2^ = 0.193. Higher opposite-gender D-scores were associated with lower opposite-gender BI scores at 100 ms. The fitted regression equation was y = −14.68x + 10.60, where x represents the opposite-gender D-score and y represents the opposite-gender 100 ms BI (Figure 4).

## 4. Discussion

The present study examined relative evaluative compatibility and attentional responses to beautified and non-beautified synthetic faces among heterosexual female university students. Three main findings emerged in relation to the proposed hypotheses. First, consistent with H1, participants showed greater relative compatibility for the beautified-positive/original-negative mapping than for the reversed mapping, although the evidence was insufficient to determine whether this compatibility effect varied by target face gender. Second, consistent with H2, the early validity effect at 100 ms was significantly larger for opposite-gender than for same-gender faces, indicating stronger early attentional orienting toward opposite-gender beautified faces. At 500 ms, the validity effect reversed numerically for opposite-gender faces, but neither this contrast nor the difference between the target face gender conditions was statistically significant. Third, the opposite-gender D-score was inversely associated with the opposite-gender 100 ms attention-bias index. The direction of this association was compatible with a habituation-based account, although the underlying mechanism could not be determined from the present data.

The inverse association between evaluative compatibility and early attentional orienting is compatible with the possibility that repeated exposure reduces the novelty and attentional priority of preferred idealized imagery. Social media provides a high-choice environment in which selective exposure and recommendation systems may increase contact with content that matches users’ preferences (Tsfati et al., 2025). Repeated encounters with beautified faces may consequently weaken responses to these stimuli through habituation (Rankin et al., 2009), particularly for mate-relevant opposite-gender faces (Shin et al., 2018). Because the 100 ms condition was intended to capture rapid attentional selection (Yan et al., 2018), reduced novelty could contribute to weaker early orienting among participants with greater relative compatibility for opposite-gender beautified faces. However, exposure history and habituation were not directly assessed, and stable differences in attentional strategy or evaluative processing remain possible alternative explanations.

Participants showed greater relative compatibility for the beautified-positive/original-negative mapping than for the reversed mapping. This pattern may partly reflect the “what is beautiful is good” stereotype, consistent with evidence that facial attractiveness influences judgments of health, kindness, intelligence, and trustworthiness (Stephen & Wei, 2015; Tsukiura & Cabeza, 2011; Gulati et al., 2024). Nevertheless, the IAT is a relative measure, and several attribute words were directly related to the target categories. The observed effect may therefore reflect both evaluative valence and semantic correspondence rather than an absolute positive evaluation of beautified faces. Although same-gender and opposite-gender faces showed a similar descriptive pattern, the interaction results did not establish moderation by target face gender. The two conditions may still involve different motivational routes, including self-relevant appearance standards for same-gender faces and mate-relevant attractiveness cues for opposite-gender faces (Hussain et al., 2025; Maner et al., 2003).

The attentional findings further showed that target face gender was associated with early attentional allocation, whereas evidence for differences at the later stage remained inconclusive. At 100 ms, the validity effect was significantly larger for opposite-gender than for same-gender faces, indicating that beautified male faces received greater priority during the early stage of processing (Bar-Haim et al., 2007). This effect may reflect the motivational relevance of attractive opposite-gender faces for heterosexual female participants, as facial attractiveness can increase the priority of potential mating-related cues (Maner et al., 2003; Thomas et al., 2020). At 500 ms, responses were numerically slower when the probe replaced the opposite-gender beautified face, a direction compatible with inhibition of return (Wang & Klein, 2010). However, the relevant validity contrast was not statistically significant, and the validity effects did not differ between the two target face gender conditions. The evidence therefore supports a target face gender difference in early orienting, whereas the later attentional pattern remains inconclusive.

Taken together, these findings suggest that relative evaluative compatibility and early attentional priority toward beautified faces do not necessarily vary in the same direction and may inform future research on digital image literacy and aesthetic education. However, the present experiment did not test any educational, cognitive, or platform-based intervention, so practical recommendations remain speculative. Three main limitations define priorities for future research. First, the modest and homogeneous sample consisted exclusively of young Han Chinese women recruited from a single university in northern China, which limits the generalizability of the findings across gender, age, ethnic, and cultural groups. Standards of facial attractiveness and responses to digitally beautified faces may vary across sociocultural contexts; therefore, the present findings should not be assumed to generalize directly to men, older adults, ethnic minority groups, or populations from other cultural settings. Second, the semantically related IAT words and the use of synthetic faces processed through a composite one-click beautification procedure constrain interpretation of the evaluative and stimulus-related effects. Third, participants’ prior exposure to beautified faces was not measured, and behavioral response times provide only an indirect account of attentional dynamics. Future studies should use larger and more diverse samples, independently selected attribute words, and more controlled or ecologically valid face stimuli; directly measure or manipulate exposure to beautified imagery; and combine behavioral tasks with eye-tracking or event-related potentials (Armstrong & Olatunji, 2012). Appearance comparison tendencies may also be examined as a potential moderator (Bonfanti et al., 2025).

## 5. Conclusions

This study examined relative evaluative compatibility, attentional responses to beautified versus non-beautified synthetic faces, and the association between these measures. Results revealed greater relative compatibility for the beautified-positive/original-negative mapping than for the reversed mapping. Evidence regarding whether this compatibility effect varied by target face gender remained inconclusive. Opposite-gender beautified faces elicited early attentional orienting at 100 ms, whereas the validity effect showed a nonsignificant reversal at 500 ms.

The opposite-gender D-score was inversely associated with the opposite-gender 100 ms BI. However, the attention-bias index employed here is subject to the well-recognized reliability challenges common to reaction-time difference measures. This individual-difference association should therefore be viewed as preliminary, warranting cautious interpretation and replication using measures with greater temporal stability. A habituation-based account offers one possible explanation for the direction of this association, but the present study did not assess exposure history or habituation directly. Future research should examine this association using measures with greater temporal precision and measurement stability and should test the habituation account by measuring or experimentally manipulating repeated exposure to beautified faces. These findings characterize relative evaluative compatibility and attentional responses within the present sample and experimental context. Replication in larger and more diverse populations is needed before broader conclusions or practical applications can be established.

## Figures and Tables

**Table 1:** IAT procedure.

Block	Function	Task Description	Trials	Response (F Key)	Response (J Key)
1	Practice	Attribute Word Identification	20	Positive Words	Negative Words
2	Practice	Target Face Identification	20	Beautified Faces	Original Faces
3	Practice	Compatible Task	20	Beautified Faces; Positive Words	Original Faces; Negative Words
4	Test	Compatible Task	60	Beautified Faces; Positive Words	Original Faces; Negative Words
5	Practice	Reversed Target Face Identification	20	Original Faces	Beautified Faces
6	Practice	Incompatible Task	20	Original Faces; Positive Words	Beautified Faces; Negative Words
7	Test	Incompatible Task	60	Original Faces; Positive Words	Beautified Faces; Negative Words

Note. The table presents the compatible-first sequence. For half of the participants, the incompatible sequence was administered before the compatible sequence. The attribute-word discrimination block was always completed first.

## Data Availability

Data, scripts, materials, and analyses are available at https://osf.io/k6wc4/ (accessed on 28 October 2025).

## Supplementary Materials

The following supporting information can be downloaded at https://www.mdpi.com/article/10.3390/bs16081421/s1, Dot-probe task image materials.

## Abbreviations

The following abbreviations are used in this manuscript:

IAT	Implicit Association Test
BF	Bayes Factor
BI	Attention-Bias Index
SOA	Stimulus Onset Asynchrony
